# In silico experimentation with a model of hepatic mitochondrial folate metabolism

**DOI:** 10.1186/1742-4682-3-40

**Published:** 2006-12-06

**Authors:** H Frederik Nijhout, Michael C Reed, Shi-Ling Lam, Barry Shane, Jesse F Gregory, Cornelia M Ulrich

**Affiliations:** 1 Department of Biology, Duke University, Durham, NC 27708, USA; 2 Department of Mathematics, Duke University, Durham, NC 27708, USA; 3 Department of Nutrition Sciences and Toxicology, University of California, Berkeley, CA 94720-3104, USA; 4 Department of Food Science and Human Nutrition, University of Florida, 32611-0370, USA; 5 Cancer Prevention Program, Fred Hutchinson Cancer Research Center, Seattle, WA 98109-1024, USA

## Abstract

**Background:**

In eukaryotes, folate metabolism is compartmentalized and occurs in both the cytosol and the mitochondria. The function of this compartmentalization and the great changes that occur in the mitochondrial compartment during embryonic development and in rapidly growing cancer cells are gradually becoming understood, though many aspects remain puzzling and controversial.

**Approach:**

We explore the properties of cytosolic and mitochondrial folate metabolism by experimenting with a mathematical model of hepatic one-carbon metabolism. The model is based on known biochemical properties of mitochondrial and cytosolic enzymes. We use the model to study questions about the relative roles of the cytosolic and mitochondrial folate cycles posed in the experimental literature. We investigate: the control of the direction of the mitochondrial and cytosolic serine hydroxymethyltransferase (SHMT) reactions, the role of the mitochondrial bifunctional enzyme, the role of the glycine cleavage system, the effects of variations in serine and glycine inputs, and the effects of methionine and protein loading.

**Conclusion:**

The model reproduces many experimental findings and gives new insights into the underlying properties of mitochondrial folate metabolism. Particularly interesting is the remarkable stability of formate production in the mitochondria in the face of large changes in serine and glycine input. The model shows that in the presence of the bifunctional enzyme (as in embryonic tissues and cancer cells), the mitochondria primarily support cytosolic purine and pyrimidine synthesis via the export of formate, while in adult tissues the mitochondria produce serine for gluconeogenesis.

## Background

Folate and one-carbon metabolism play a central role in cellular physiology because they are intimately involved in the control of purine, pyrimidine, and glutathione synthesis, as well as the methylation of DNA, histones and a host of other key cellular components. Deficiencies in folate metabolism have been associated with a wide range of diseases and pathologies such as anemia, spina bifida, cancer, cardiovascular disease, and neuropsychiatric disorders. Aberrant folate metabolism can be caused by polymorphisms in the genes for enzymes in the folate and methionine cycles, environmental factors that increase oxidative stress, and dietary deficiencies in B vitamins. Thus, this part of cell metabolism is a locus where genetic, environmental, and behavioral variables interact to affect many aspects of health and disease [1-12].

**Figure 1:**
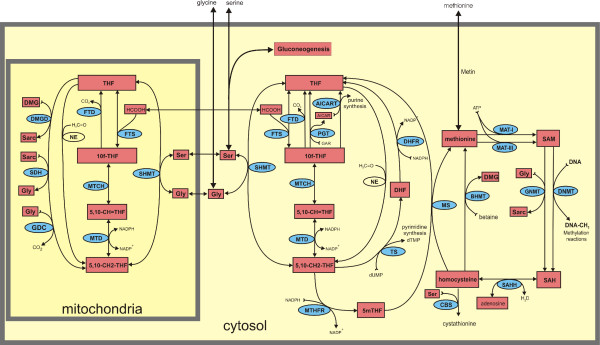
Diagram of the reactions modeled in the present paper. Pink rectangles represent variable metabolites and blue ellipses are enzymes. Full names corresponding to acronyms are given in Additional file 1.

It has been known for almost 50 years that some reactions of the folate cycle in eukaryotes (Figure 1) are duplicated in the cytosol and mitochondria [13], while other reactions such as purine and pyrimidine synthesis occur only in the cytosol, and the glycine cleavage system occurs only in the mitochondria [14,15]. Two folate substrates, dihydrofolate (DHF) and 5-methyltetrahydrofolate (5mTHF), occur only in the cytosol. Moreover, some enzymes of mitochondrial folate metabolism are highly up-regulated in embryos and cancer cells and virtually inactive in normal adult cells [16,17].

Because substrates, enzymes, and function differ between the mitochondrial and cytosolic compartments, many questions arise. What specific role does the mitochondrial folate cycle play in overall cell metabolism? What is the reason for the down-regulation of mitochondrial MTD and MTCH in adult tissues? Why does SHMT occur in both compartments? What is the role of the mitochondrial GDC reaction? How does the system accommodate changes in the input of serine and glycine? What happens during protein or methionine loading? What are the roles of folate-binding proteins in the cytosol and the mitochondria? These questions have been the subject of extensive experimental investigation and theoretical discussions [16-26].

We have developed a mathematical model for mitochondrial and cytosolic one-carbon metabolism. The model extends our earlier models of cytosolic methionine and folate metabolism [27-30]. We use the model to conduct *in silico *experiments that address many of the above questions and compare the results to experimental observations. The model gives insights into the mechanisms underlying the experimental results and allows us to test various hypotheses that have been proposed in the literature.

In the following section we give a brief overview of our model. Full details of the model and the full names of all abbreviations used in the text and in the model are given in additional file 1.

### Model overview

Figure 1 shows the biochemical pathways in the hepatic cellular model used in this paper. Rectangular boxes represent the substrates that can vary in the model, and the ellipses contain the acronyms of the enzymes that catalyze specific reactions. Full names of the substrates and enzymes appear in the Additional File 1. Non-boxed substrates are taken to be constant. The model consists of 23 coupled differential equations for the time courses of the boxed substrates. The formulae for the velocities of the various reactions are taken, when possible, from the experimental literature. In some cases we adjusted the rate constants within experimental ranges so the concentrations of folates in the cytosol and mitochondria would be similar to those observed experimentally. In this model, MTD, MTCH, FTD and FTS are active in the mitochondria, and TS and DHFR are up-regulated in the cytosol, so the model represents a liver cell that is actively dividing.

This model is an extension of the model used by Reed et al. [30]. Mitochondrial folate substrates and enzymes were added, and sarcosine and dimethylglycine are new variables (each is assumed to have the same concentrations in the cytosol and mitochondria). The extracellular serine, glycine, and methionine concentrations can be specified as functions of time. The cytosolic and mitochondrial serine and glycine concentrations can vary, as can the cytosolic methionine concentration. Appropriate kinetics are used for transport between the external, cytosolic, and mitochondrial compartments. The mitochondrial and cytosolic HCOOH concentrations are allowed to vary in the present model, and formate is transported between the mitochondrial and cytosolic compartments. In addition, AICAR is a variable in the model that couples the PGT and AICART reactions. Finally, we have added a serine sink that corresponds to the use of cytosolic serine in gluconeogenesis and the tricarboxylic acid cycle. Full details of the model are given in Additional file 1.

Table 1 shows the steady-state concentrations and velocities in the model assuming that the extracellular glycine, serine, and methionine concentrations are held constant at 300 μM, 150 μM, and 30 μM respectively. For the reversible reactions in Figure 1, the positive directions are as follows: 5,10-CH_2_-THF to 5,10-CH = THF to 10f-THF for MTD and MTCH; THF to 10f-THF for FTS; serine to glycine for SHMT; SAH to homocysteine for SAAH. We refer to the values shown in Table 1 as "normal" throughout this paper. The computed distributions of folates in the cytosol and mitochondria are similar to those reported by Cook [15].

**Table 1:** Cytosolic and mitochondrial concentrations and velocities at steady-state.

Cytosolic				Mitochondrial			
	
Concentrations (μM)		Velocities (μM/h)		Concentrations (μM)		Velocities (μM/h)	
THF	3.74	MTD	-113	THF	20.8	MTD	2106
5,10-CH = THF	0.26	MTCH	-113	5,10-CH = THF	1.55	MTCH	2106
5,10-CH2-THF	0.46	FTS	546	5,10-CH2-THF	1.7	FTS	-1639
10f-THF	3.23	FTD	69.4	10f-THF	16.0	FTD	467
5mTHF	5.55	SHMT	9.2			SHMT	84.3
DHF	0.035	NE	46.1			NE	279
Aicar	0.95	AICART	182			GDC	1560
		DHFR	120			SDH	122
		TS	120			DMGD	60.7
Ser	551	MTHFR	47.7	Ser	1963		
Gly	830	PGT	182	Gly	1858		
HCOOH	14.5	MS	47.7	HCOOH	59.9		
Met	51.9	BHMT	60.7				
SAM	63.6	CBS	99.7				
SAH	13.1	DNMT	147				
Hcy	1.10	GNMT	61.4				
		MAT-I	132				
		MAT-III	76.1				

In the model, we express the quantities of substrates by concentrations and we assume that the mitochondria occupy 1/4 of the cell volume. So the same number of molecules will have different concentrations in the two compartments. For example, we assume that the total "normal" cellular folate concentration is 20 μM. If the folate molecules are equally divided between the cytosol and the mitochondria [15], then the total folate concentrations will be 13.3 μM and 40 μM in the cytosol and the mitochondria, respectively. We assume that sarcosine and dimethylglycine diffuse freely and thus have the same concentrations in the cytosol and mitochondria. For transport between compartments, the rates (in μM/h) are the rates of change of concentration in the cytosol. If a substrate has concentration C_c _in the cytosol and concentration C_m _in the mitochondria, then the observed total cellular concentration (when the compartments are combined) will be (0.75)C_c _+ (0.25)C_m_. Table 2 shows the normal total cellular concentrations of the folate metabolites and amino acids.

**Table 2:** Total cellular concentrations of folate metabolites and amino acids at steady-state

Metabolite	Concentration (μM)
THF	8.02
5,10-CH = THF	0.58
5,10-CH2-THF	0.77
10f-THF	6.43
5mTHF	4.16
DHF	0.026
	
Sarcosine	9.11
Dimethylglycine	0.70
Serine	904
Glycine	1087

Table 3 shows the normal rates of transport of serine, glycine, and HCOOH, between the compartments. For example, at normal steady-state, the cytosol receives 799 μM/h of serine from the extracellular medium and loses 30.4 μM/h to the mitochondria (Table 3). Of course, the cytosol also loses 99.7 μM/h through the CBS reaction, 8.19 μM/h through the cytosolic SHMT reaction, and 662 μM/h to gluconeogenesis (Tables 1 and 3).

**Table 3:** Transport rates at steady-state (μM/h).

	Serine	Glycine	HCOOH
Extracellular to cytosol	799	504	--
Mitochondria to cytosol	-28.1	-451	546
To gluconeogenesis	662		

### Limitations of the model

The model we developed is intended specifically to study the interaction among the folate cycles in the cytosol and mitochondria. No mathematical model can include the complete biological complexity of a system. Instead, a model should contain sufficient detail to allow investigators to study the phenomenon of interest without omitting features that are likely to affect the behavior of the model dramatically. The model used in this paper, and described in detail in Additional File 1, is no exception. For instance, we do not include leucovorin as a cytosolic folate substrate, we do not include the polyamine pathway, and we use only two methyltransferase reactions between SAM and SAH. We also do not include the fact that some folate substrates can regulate gene expression of some folate enzymes. We do not include the allosteric binding of folates to folate enzymes, because the main effect of these reactions is to maintain reaction velocities in the face of severe folate deficiency (studied in Nijhout et al. [28]). Our model assumes that cytosolic amino acids are used only as substrates in the folate and methionine cycles, and as a source for gluconeogenesis. We do not include protein catabolism as a source, or protein synthesis as a sink, for amino acids. Likewise, we do not include the transport of homocysteine between the blood and the cellular compartment. All these features are important and interesting aspects of folate metabolism, and several are currently under investigation, but they do not bear directly on the role of mitochondrial folate metabolism studied in this paper.

## Results

### A. Variation in serine and glycine

There is general agreement that one of the functions of the mitochondrial folate cycle is to provide 1-C units to the cytosol for purine and pyrimidine synthesis and the methylation reactions in rapidly dividing cells [14-16]. The two primary sources of 1-C units are the mSHMT reaction, which converts serine to glycine, and the GDC reaction, which breaks down glycine, producing 5,10-methyleneTHF. Additional contributions are made by the SDH and the DMGD reactions. All four of these reactions use THF to produce 5,10-methyleneTHF, which is converted to 10f-THF. The FTS reaction regenerates THF and produces free formate that is exported to the cytosol. There are a number of natural questions here. (1) How does this system operate at different levels of extracellular serine and glycine? (2) How sensitive is the production of formate to the balance between serine and glycine? (3) How sensitive is the production of purines and pyrimidines in the cytosol to the supply of formate?

**Figure 2:**
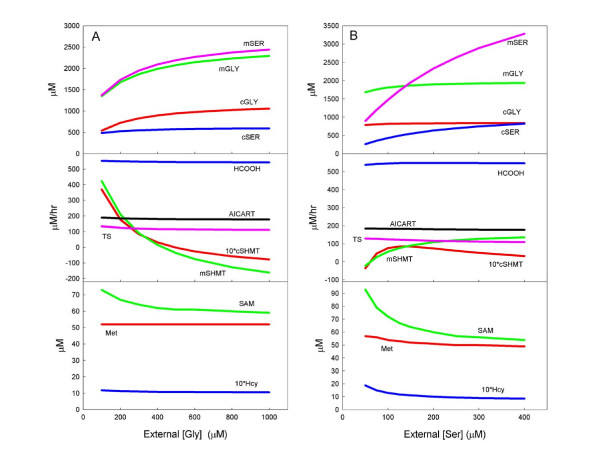
A: Response of selected model variables to variation in external glycine input. B: Response of model variables to variation in external serine input.

To investigate these questions, we systematically altered the external concentrations of serine and glycine from their normal values of 150 μM and 300 μM, respectively. Figure 2A shows the cytosolic and mitochondrial glycine and serine concentrations as the extracellular glycine concentration is varied from 100 μM to 1000 μM. As external glycine increases, both the cytosolic and mitochondrial glycine concentrations increase, but not as dramatically, because the reverse transport out of the mitochondria and cytosol increases as the concentrations rise. The cytosolic serine concentrations also rise because of the interconversion of glycine and serine by the SHMT reactions.

As external glycine rises, both the cytosolic and the mitochondrial SHMT reactions reverse and run in the glycine to serine direction (Fig. 2A); the mitochondrial reaction is more sensitive to external glycine. This reversal was observed by Kastanos, Woldman, and Appling [21], who grew yeast in pure serine and pure glycine environments. In spite of great variations in the glycine and serine concentrations (Fig. 2A, middle panel, blue curve) and the reversals in both SHMT reactions, the rate at which formate is supplied to the cytosol remains remarkably constant (Fig. 2A). In addition, the rates of the TS and AICART reactions (for thymidylate and purine synthesis, respectively) change very little despite large changes in external glycine. The metabolites of the methionine cycle also change relatively little except for SAM, which rises at low external glycine because the GNMT reaction rate declines (Fig. 2A).

Figure 2B shows the cytosolic and mitochondrial serine and glycine concentrations as the extracellular serine concentration is varied from 50 μM to 400 μM. As external serine increases, both the cytosolic and mitochondrial glycine concentrations increase, and there is an even greater increase in the cytosolic and mitochondrial serine concentrations. As external serine decreases from normal, both the cytosolic and the mitochondrial SHMT reactions reverse and run in the glycine to serine direction (Fig. 2B); as before, the mitochondrial reaction is more sensitive. The rates of the TS and AICART reactions and the rate of transport of formate out of the mitochondria are again remarkably stable.

The methionine cycle metabolites are more sensitive to external serine than to external glycine, especially at low external serine. This is because serine is required for the CBS reaction, which slows down and causes methionine cycle metabolites to accumulate, particularly as SAM, owing the internal regulatory mechanisms of the methionine cycle [27,29].

Lewis et al. [18] radiolabeled the methyl group of SAM. Very little of the radioactivity appeared in metabolites of the folate and methionine cycles because most of these radiolabeled methyl groups were transferred to other substrates by the methylation reactions. However, when glycine was elevated, the amount of radiolabel in HCOOH, serine, and CO_2 _went up considerably. This can be easily explained by the model. More glycine results in acceleration of the GNMT reaction so more of the radiolabeled methyl groups are put into sarcosine. Then, in the mitochondria, the sarcosine becomes either radiolabeled 5,10-CH_2_-THF or CO_2_. The radiolabeled 5,10-CH_2_-THF makes radiolabeled HCOOH via the MTD, MTCH, and FTS reactions and radiolabeled CO_2 _via the FTD reaction. High glycine increases the glycine-to-serine rate of the SHMT reactions in both cytosol and the mitochondria, so more radiolabel appears in serine.

### B. Reduced folate status

**Figure 3:**
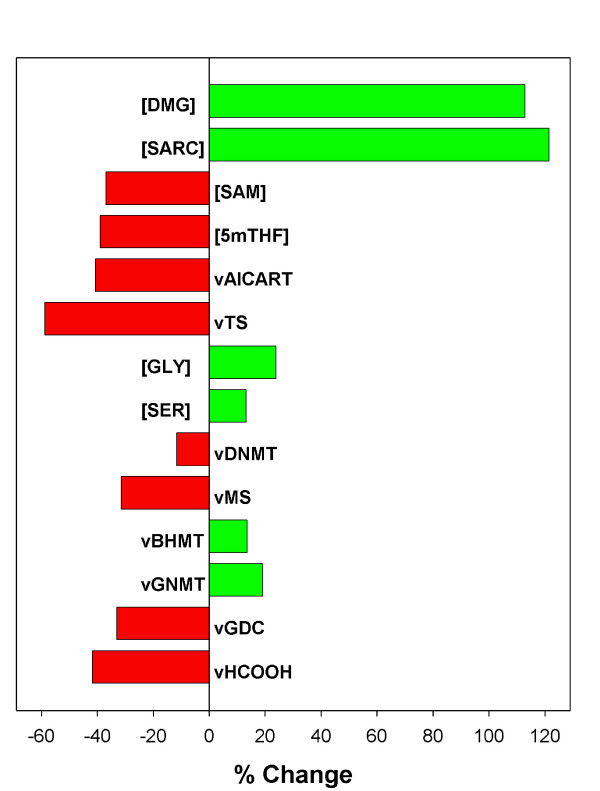
Change in the steady-state concentrations of selected metabolites and steady-state reaction velocities of selected reactions in the presence of a 50% reduction in folate levels.

Figure 3 shows the percentage change in the steady-state concentrations or rates of various metabolites and reactions in the presence of a 50% reduction in folate levels. The concentration of 5mTHF drops, of course, and this releases the inhibition of GNMT so the GNMT reactions goes faster and depletes SAM. Methionine and SAM are also depleted because less homocysteine is remethylated. Because of the long-range regulations in the methionine cycle [29], the rate of the DNMT methylation reaction decreases only modestly. The transport of formate from the mitochondria decreases and thymidylate synthesis and purine synthesis rates are reduced dramatically. The rate of the GDC reaction in the mitochondria drops because mitochondrial THF is much lower. Since the GDC reaction catabolizes glycine, the concentration of glycine rises, which also drives up the concentration of serine via the SHMT reaction. It was observed by Allen et al. [31] that both sarcosine and dimethylglycine are elevated in patients with folate deficiency, clinical results that we observe also in the model (Figure 3). More sarcosine is produced in the cytosol because the GNMT rate is elevated (because there is less inhibition by 5mTHF), and more dimethylglycine is produced because the rate of the BHMT reaction is elevated. Since the concentration of THF is lower, sarcosine and dimethylglycine are used at lower rates in the mitochondria.

### C. SHMT expression

MacFarlane et al. [32] report that mice lacking liver cytosolic SHMT have a normal SAM/SAH ratio. To test this in the model, we eliminated the cytosolic SHMT reaction entirely. This resulted in a steady-state of [SAM] = 62.6 μM, [SAH] = 13.1 μM. Normal values of these metabolites are in the model [SAM] = 63.6 μM, [SAH] = 13.1 μM. This is not surprising because the normal net rate of the cSHMT reaction is very low (9.2 μM/h), as this enzyme is poised to modulate fluctuations in serine and glycine by interconverting them.

Herbig et al. [14] found that increasing the expression of cSHMT lowers the SAM concentration in a glycine-dependent manner. They suggested two possible alternative mechanisms: (1) that cSHMT is in competition with MTHFR for 5,10-CH_2_-THF, and therefore higher SHMT expression should lower [5mTHF] and thus lower the rate of remethylation of homocysteine to methionine, lowering SAM; or (2) that cSHMT sequesters 5mTHF, lowering its free concentration and thereby lowering SAM as in (1). In the current model, if SHMT does not bind to 5mTHF, we found that the SAM concentration is quite insensitive to the amount of cSHMT and also insensitive to the external glycine concentration, which does not support the first hypothesis. When we add the binding of 5mTHF to SHMT to the model, and then up-regulate cSHMT, we find that the SAM concentration is substantially reduced, supporting the second hypothesis (simulations not shown).

Several authors have observed that Chinese hamster ovary (CHO) cells that lack mSHMT are glycine auxotrophs (see, for example, [32,33]). These findings have been interpreted as indicating that mitochondria normally supply glycine to the cytosol. The model suggests that the situation is more complicated and more interesting. In the model, under normal conditions in hepatic cells, there is a net influx of glycine into the mitochondria (Table 4). If we eliminate the mSHMT reaction, there is a modest decrease in cytosolic and mitochondrial glycine, but the net flux remains from cytosol to mitochondria. On the other, if mSHMT is normal and we set the external glycine to zero, the concentrations of glycine in the cytosol and mitochondria decline to about one quarter of their normal value but not to zero. In addition, the mitochondria become net exporters of glycine to the cytosol (Table 4). This is because the velocity of mSHMT reaction from serine to glycine increases 10-fold. However, if mSHMT is eliminated *and *the external glycine is set to zero, then the glycine concentration declines to almost one tenth its normal value and formate production by the mitochondria is cut in half. Thus the elimination of mSHMT and external glycine together is clearly very detrimental to the cell. Interestingly, the net flux of glycine remains from cytosol to mitochondria. If one adds glycine back into the external medium the cell becomes almost normal (Table 4).

**Table 4:** Effects of variation in mSHMT and external glycine*

	Normal	mSHMT = 0	Extenal gly = 0	mSHMT = 0 Extenal gly = 0
cellular glycine	1087	1046	272	123
flux of glycine from mitochondria to cytosol	-451	-474	+90	-67
velocity of mSHMT	84	0	868	0
velocity of cSHMT	9.2	19	114	218
flux of HCOOH to cytosol	546	524	538	245

*Concentrations are in μM and fluxes and velocities are in μM/h.

### D. The GDC reaction

**Figure 4:**
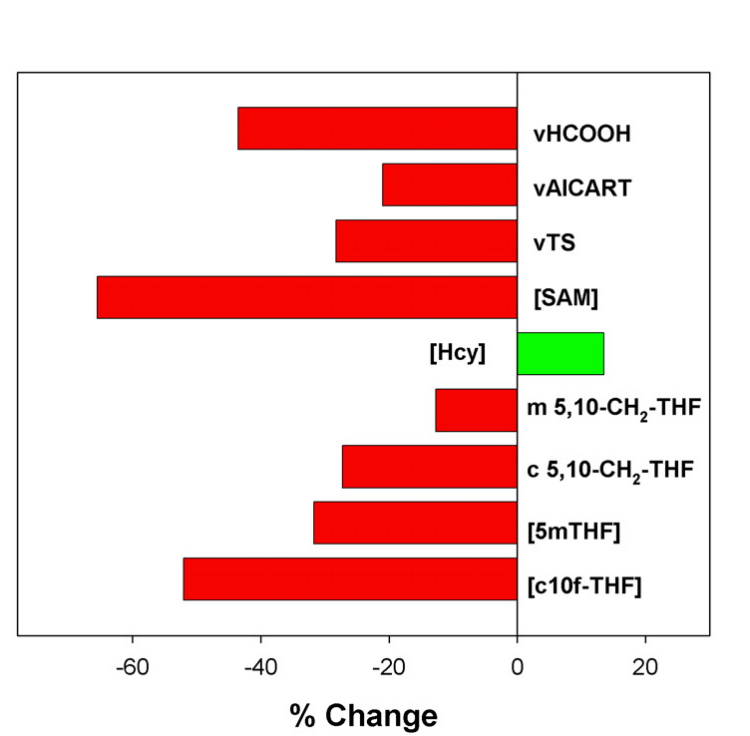
Change in the steady-state concentrations of selected metabolites and steady-state velocities of selected reactions when the GDC reaction is eliminated.

In order to study the contribution of the GDC reaction, we set its velocity to zero in the model and calculated the percentage change in concentrations and fluxes at the new steady state (Figure 4). Because the GDC reaction is turned off, the concentration of 5,10-CH_2_-THF in the mitochondria drops dramatically, which lowers the flux through the MTD, MTCH, and FTS reactions. Thus, much less formate is produced in the mitochondria and therefore the rate of export of formate to the cytosol declines to about 50% of its former value. Because of the reduced supply of formate, the concentration of cytosolic 10f-THF goes down. This has two effects. First, fewer purines are produced and second, the net flux from 10f-THF to 5,10-CH_2_-THF reverses so that the net flux is from 5,10-CH_2_-THF to 10f-THF. This reduces the cytosolic concentration of 5,10-CH_2_-THF, which causes thymidine synthesis to drop. It also makes the concentration of 5mTHF drop, which causes the homocysteine concentration to rise. The decline in [5mTHF] releases the inhibition of GNMT, which draws down [SAM].

### E. Eliminating the mitochondrial bifunctional enzyme

**Figure 5:**
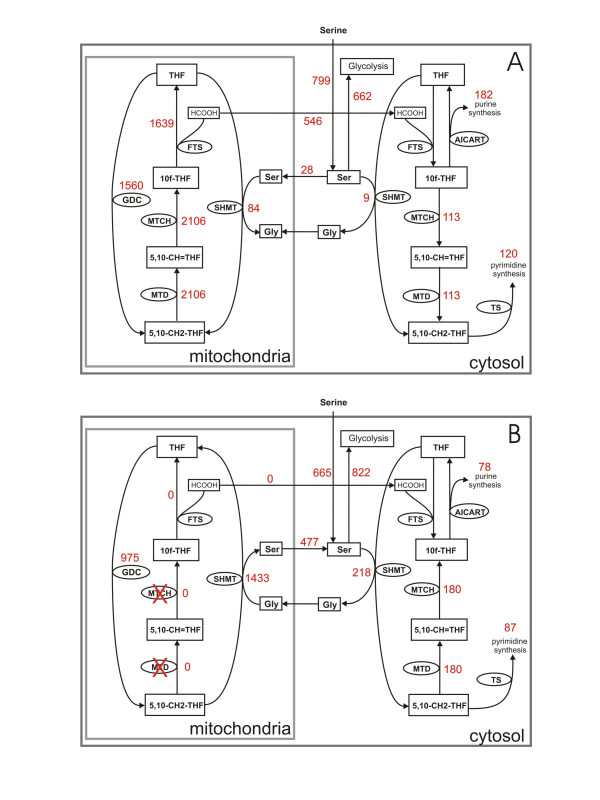
Effect of eliminating the mitochondrial bifunctional enzyme. A: selected reaction velocities when bifunctional enzyme is active. B: reaction velocities when bifunctional enzyme is eliminated. Reaction velocities are indicated with red numbers, and the units are μM/h.

The mitochondrial bifunctional enzyme is active during embryonic development and in transformed cells [17,24], but not in the adult liver. We examined the effect of eliminating the bifunctional enzyme by setting the V_max _of the mitochondrial MTD and MTCH reactions to zero. The significant changes in mitochondrial and cytosolic one-carbon metabolism are shown in Figure 5. The mitochondrial GDC reaction slows down somewhat, and the mSHMT reverses dramatically in the direction of serine production. Formate production by the mitochondria is reduced to zero. Thus the mitochondria have switched from being formate factories to being serine factories.

In the cytosol, thymidylate and purine synthesis are markedly reduced, and the cMTD and cMTCH reactions reverse and now run strongly in the direction from 5,10-CH_2_-THF to 10f-THF. The export of serine to gluconeogenesis is greater than the serine import into the cell from the blood (the opposite is true when the bifunctional enzyme is present). Thus, when the bifunctional enzyme is present (the case we call normal), mitochondrial folate metabolism produces formate for the cytosol for purine and pyrimidine synthesis and methylation reactions. When the bifunctional enzyme is absent, mitochondrial folate metabolism produces serine for the cytosol and overall folate metabolism is a net producer of serine for gluconeogenesis.

### F. Eliminating the mitochondria

**Figure 6:**
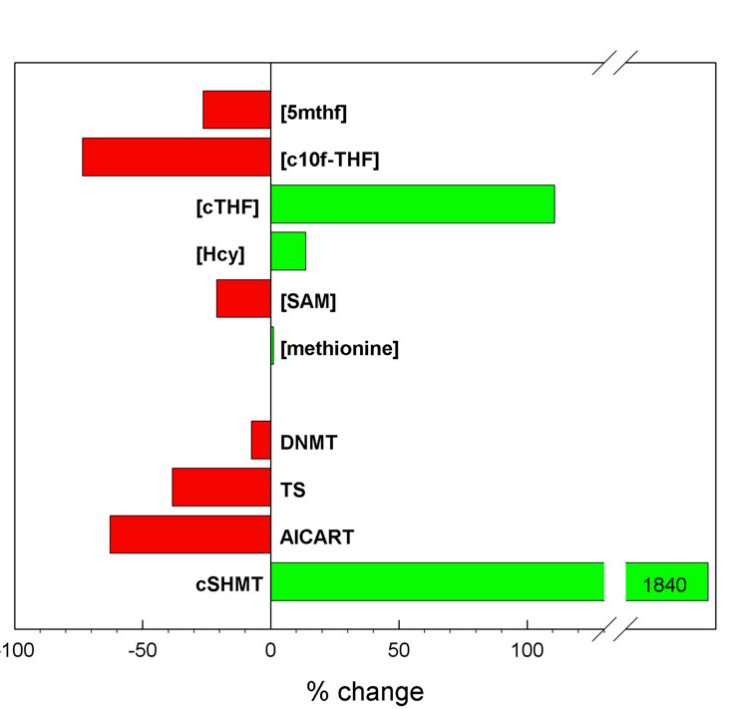
Change in the steady-state concentrations of selected metabolites and steady-state reaction velocities of selected reactions when the mitochondrial reactions are eliminated form the model.

We examined the significance of the mitochondrial folate cycle for overall one-carbon metabolism by eliminating the mitochondrial folate reactions entirely (setting all the velocities to zero). The significant changes in cytosolic one-carbon metabolism are indicated in Figure 6. There is now no formate at all being supplied by the mitochondria so the rates of thymidylate synthesis and purine synthesis are greatly reduced. The rate of the cytosolic SHMT increases approximately 18-fold and the cytosolic MTD and MTCH reactions reverse and now run in the direction from 5,10-CH_2_-THF to 10f-THF (not shown). The cytosolic concentration of 5,10-CH_2_-THF drops, causing the concentration of 5mTHF to drop, which in turn causes homocysteine to rise. The concentrations of methionine and SAM are not much affected. It is interesting to observe that the elimination of the mitochondrial folate metabolism does not completely disrupt cytosolic folate metabolism; in fact the only major effects are on the rates of thymidylate and purine synthesis, while the behavior of the methionine cycle (including the DNMT reaction) is largely unaffected. This is consistent with the hypothesis that the main role of mitochondrial folate metabolism is to supply extra 1-carbon units to the cytosol as formate [16,17,24].

### E. Methionine and protein loading

**Figure 7:**
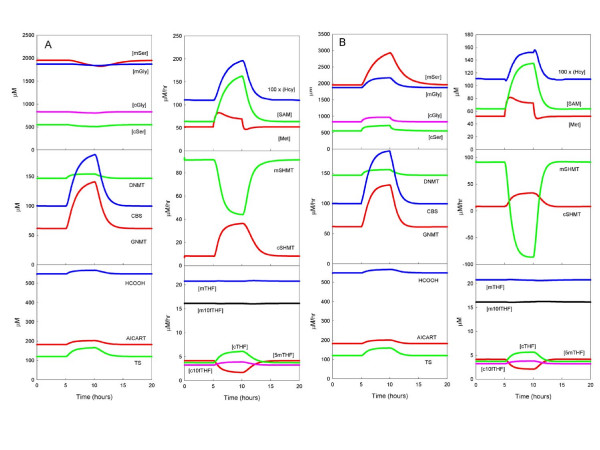
A: Response of selected model variables to a 5-hour pulse of elevated external methionine. B: Response to a 5-hour pulse of elevated external methionine, glycine and serine. The pulses consisted of a doubling of the external amino-acid concentrations from 5 to 10 hours after initiation of the simulation.

The mathematical model allows us not only to compute steady states but also to compute the time course of concentrations and fluxes as they respond dynamically to changing inputs. We examined how the system responded to a methionine load, which we simulated by doubling the external methionine concentration from 30 to 60 μM during hours 5–10 of a 20 hour simulation (Figure 7A). As expected [27,29], methionine rises modestly, SAM rises substantially, but the DNA methylation rate is very stable because the extra methyl groups are carried by an increase in the rate of the GNMT reaction during loading. The rise in SAM also causes an increase in the rate of the CBS reaction during loading, so there is an increased removal of serine from the system, which causes both cytosolic and mitochondrial serine concentrations to decrease. Despite the increase in the rate of the CBS reaction, homocysteine almost doubles because SAM is inhibiting BHMT and MTHFR, which decreases 5mTHF and slows the MS reaction. Purine and thymidylate synthesis rise modestly during loading because of modest increases in cytosolic 10f-THF and 5,10-CH_2_-THF, and there is a decline of 5mTHF accompanied by an increase in cytosolic THF. The mitochondrial folates are virtually unchanged, as is the rate of transport of formate from the mitochondria to the cytosol.

To simulate a protein meal, we not only doubled methionine for five hours but also doubled the external serine and glycine concentrations for the same five hours (Figure 7B). The major difference is that all the serine and glycine concentrations rise during protein loading and the mitochondrial SHMT reaction reverses direction. The increase in serine increases the CBS reaction and the fraction transsulfurated, and the increase in glycine increases the GNMT reaction. Both of these changes prevent SAM from rising as high as it did under a pure methionine load. In turn, the inhibition of MTHFR by SAM is diminished, so 5mTHF does not fall as much as during a methionine load. The joint effect is that homocysteine does not rise nearly as much as during a pure methionine load. Purine and thymidylate synthesis rise modestly during protein loading because of modest increases in cytosolic 10f-THF and 5,10-CH_2_-THF. Compared to methionine loading, there are smaller changes in the cytosolic folates and, as above, the rate of transport of formate from the mitochondria to the cytosol is remarkably constant.

## Discussion

Understanding the function of the compartmentalization of the folate cycle between the cytosol and mitochondria poses many challenges. Measuring concentrations and especially reaction velocities is not easy in living cells, and these measurements become particularly difficult when substrates and some reactions are sequestered into different compartments. It is especially challenging, and in many cases impossible, to measure several different variables at the same time and to track changing concentrations or velocities over time. A mathematical representation of the substrates and reactions of folate metabolism allows one to conduct *in silico *experiments to test ideas and hypotheses about how the system as a whole operates. Of course, no such mathematical model can represent the complete physical and biological complexity of a real cell, and the usefulness of the model depends on how accurately it represents the known biology of the cell. Experiments with the mitochondrial model in this paper show that its behavior is consistent with a wide body of experimental findings.

Among the important questions investigated or discussed by experimentalists in recent years are: the role of the mitochondrial bifunctional enzyme, the effect of cytosolic and mitochondrial SHMT expression on purine and pyrimidine synthesis, the directionality of the SHMT reactions, the relative roles of serine and glycine as one-carbon donors, the effects of protein and methionine loading, the significance of the GDC reaction and, indeed, the significance of the mitochondria themselves. Our mathematical model has allowed us to explore these questions by *in silico *experimentation and we hope thereby to shed light on these questions and to contribute to the ongoing discussion.

Particularly interesting is the remarkable stability of formate production in the mitochondria in the face of large changes in serine and glycine input. As a consequence, the cytosol has an almost constant input of formate for purine and pyrimidine synthesis, despite short term and long term variations in glycine and serine availability. This effect is largely due to the efficient rebalancing of the serine and glycine concentrations by the reversible mitochondrial and cytosolic SHMT reactions.

Another highlight that we found is that eliminating the mitochondrial bifunctional enzyme did not lead to a runaway accumulation of 5,10-CH_2_-THF in the mitochondria as might be expected. Instead, the mitochondrial SHMT reaction reverses direction strongly and the mitochondria become net exporters of serine. Thus, in the presence of the bifunctional enzyme (as in embryonic tissues and cancer cells), the mitochondria primarily support cytosolic purine and pyrimidine synthesis via the export of formate, while in adult tissues the mitochondria produce serine for gluconeogenesis.

We plan to continue our investigations by conducting a complete fluctuation analysis [29] of the stability of formate production and purine and pyrimidine synthesis. In addition, we will continue to use the model to explore other important aspects of folate biochemistry and compartmentalization by studying the roles of folate-protein binding, substrate channeling, and the interactions of simultaneous genetic polymorphisms with variation in dietary input and vitamin status. We plan to investigate the dynamic properties and potential interactions among the many methyltransferases that act in parallel using SAM as a substrate. Finally, we plan to investigate the dynamic transport processes that determine the compartmentalization of the metabolites of folate and methionine cycles between the liver and the blood.

## Abbreviations

The attached Additional File 1 contains a complete description of the mathematical model as well as full names of all abbreviations used in the text and in the model.

## Competing interests

The author(s) declare that they have no competing interests.

## Authors' contributions

HN, MR, BS, JG and CU participated in the formulation of the questions and made substantial contributions to the design of the project and revised the intellectual content of the manuscript. HN, MR and SL wrote the code and carried out the *in silico *experimentation. HN and MR wrote the first draft of the manuscript. All authors read and approved the final manuscript.

## Supplementary Material

Additional File 1 contains a complete description of the mathematical model as well as full names of all abbreviations used in the text and in the model. Click here for file
