# Supplementary material for: In silico experimentation with a model of hepatic mitochondrial folate metabolism
Source: Theor Biol Med Model. 2006 Dec 6;3:40. doi: 10.1186/1742-4682-3-40 (PMC1713227; doi:10.1186/1742-4682-3-40)
Supplement: Additional File 1 — contains a complete description of the mathematical model as well as full names of all abbreviations used in the text and in the model. [file 1742-4682-3-40-S1.pdf]

Supplementary Material – Model Details

for

In Silico Experimentation with a Model of Hepatic Mitochondrial Folate Metabolism

H. Frederik Nijhout [1]*, Michael C. Reed[2], Shi-Ling Lam[1], Barry Shane[3], Jesse F. Gregory III[4], Cornelia M. Ulrich[5]

[1] Department of Biology, Duke University, Durham, NC 27708

[2] Department of Mathematics, Duke University, Durham, NC 27708

[3] Department of Nutrition Sciences and Toxicology , University of California, Berkeley, 94720

[4] Department of Food Science and Human Nutrition, University of Florida, 32611-0370

[5] Fred Hutchinson Cancer Research Center, Seattle, WA 98109-1024.

* Correspondence to: H. Frederik Nijhout,Department of Biology, Duke University, Durham, NC 27708, Tel. 919-684-4223; Fax. 919-660-7293; E-Mail: hfn@duke.edu.

The model consists of 23 differential equations that express the rate of change of each of the substrates in the rectangular boxes in Figure 1. Each of the differential equations is a mass balance equation that says that the time rate of change of the particular metabolite is the sum of the rates at which it is being made minus the rates it is being consumed in biochemical reactions plus or minus net transport rates from other compartments. In order to display the differential equations in a coherent and understandable way, we have chosen notation for the variables and reaction rates that is both more uniform and more spare than some notation commonly in use, for example the concentration of 5-methyltetrahydrofolate is denoted 5mf instead of the usual [5mTHF]. All of this notation is described in Part A, below. In Part B we give the differential equations, which are written in terms of reaction and transport rates. In Part C the kinetic formulas and constants for these reaction and transport rates are given with some justifications. Part D describes how the model works, what is given and what is computed, in particular experiments.

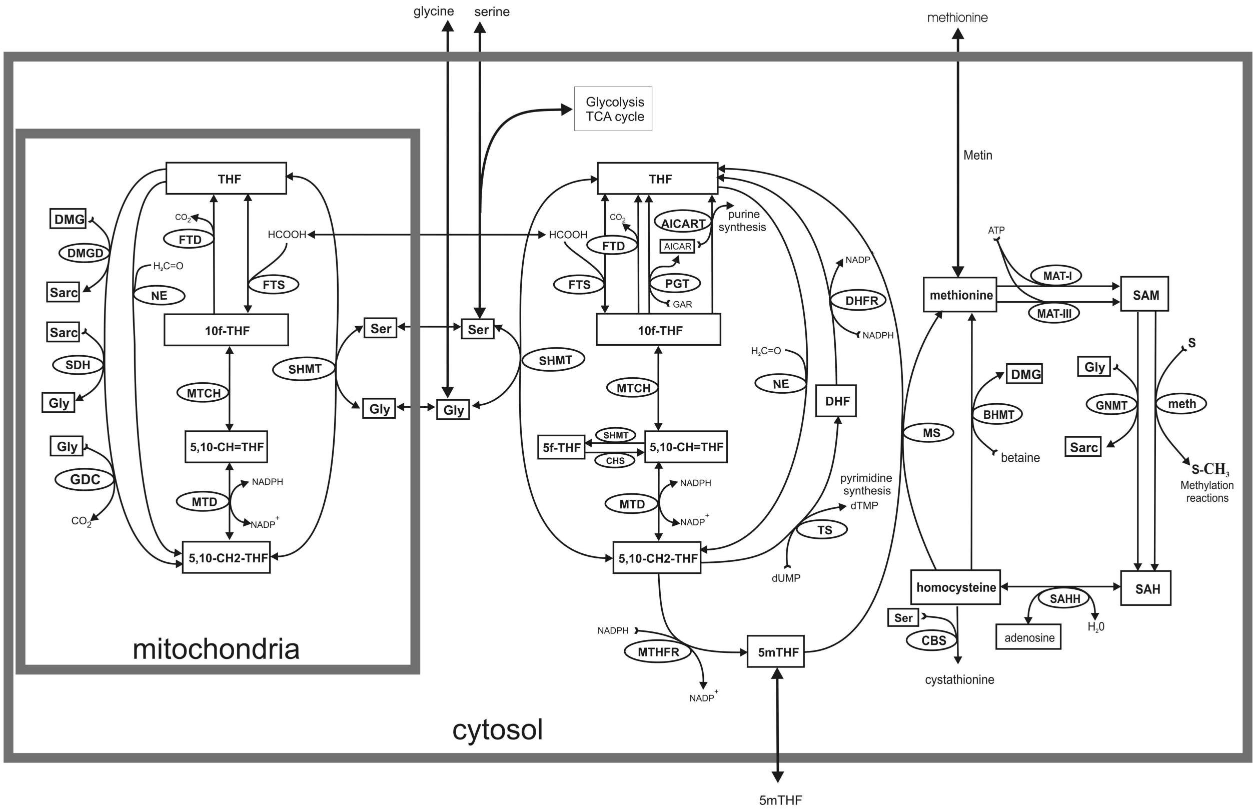

Figure 1: **Folate and Methionine Metabolism.**

Part A: Notation.

The complete names of the enzymes indicated by acronyms in Figure 1 are as follows.

Table S1: Enzyme names and acronyms.

**mitochondrial folate cycle enzymes**	
$_{m}$SHMT	serinehydroxymethyltransferase
$_{m}$FTD	10-formyltetrahydrofolate dehydrogenase
$_{m}$FTS	10-formyltetrahydrofolate synthase
$_{m}$MTCH	5,10-methenyltetrahydrofolate cyclohydrolase
$_{m}$MTD	5,10-methylenetetrahydrofolate dehydrogenase
$_{m}$NE	non-enzymatic conversion
DMGD	dimethylglycine dehydrogenase
SDH	sarcosine dehydrogenase
GDC	glycine decarboxylase (glycine cleavage system)
**cytosolic folate cycle enzymes**	
$_{c}$SHMT	serinehydroxymethyltransferase
DHFR	dihydrofolate reductase
$_{c}$FTD	10-formyltetrahydrofolate dehydrogenase
$_{c}$FTS	10-formyltetrahydrofolate synthase
$_{c}$MTCH	5,10-methenyltetrahydrofolate cyclohydrolase
$_{c}$MTD	5,10-methylenetetrahydrofolate dehydrogenase
MTHFR	5,10-methylenetetrahydrofolate reductase
AICART	aminoimidazolecarboxamide ribonucleotide transferase
PGT	Phosphoribosyl glycinamidetransformalase
$_{c}$NE	non-enzymatic conversion
TS	thymidylate synthase
**methionine cycle enzymes**	
MAT-I	methionine adenosyl transferase I
MAT-III	methionine adenosyl transferase III
GNMT	glycine N-methyltransferase
DNMT	DNA-methyltransferase
SAAH	S-adenosylhomocysteine hydrolase
MS	methionine synthase
BHMT	betaine-homocysteine methyltransferase
CBS	cystathionine β-synthase

We will use lower case three letter abbreviations for the concentrations of metabolites (μM). A prefix of $_{m}$, $_{c}$, or $_{b}$, for mitochondria, cytosol, or blood, indicates the compartment. Metabolites occuring in only one compartment (like met), or metabolites whose concentrations are assumed equal in different compartments (like dmg and src) have no prefixes.

Table S2: Names of variables (μM).

**mitochondrial folate cycle metabolites**	
$_{m}$thf	tetrahydrofolate
$_{m}$2cf	5-10-methylenetetrahydrofolate
$_{m}$1cf	5-10-methenyltetrahydrofolate
$_{m}$10f	10-formyltetrahydrofolate
**cytosolic folate cycle metabolites**	
$_{c}$thf	tetrahydrofolate
$_{c}$2cf	5-10-methylenetetrahydrofolate
$_{c}$1cf	5-10-methenyltetrahydrofolate
$_{c}$10f	10-formyltetrahydrofolate
dhf	dihydrofolate
5mf	5-methyltetrahydrofolate
aic	P-ribosyl-5-amino-4-imidazole carboxamide
**methionine cycle metabolites**	
met	methionine
sam	S-adenosylmethionine
sah	S-adenosylhomocysteine
hcy	homocysteine
**other metabolites**	
$_{m}$gly	mitochondrial glycine
$_{m}$ser	mitochondrial serine
$_{m}$COO	mitochondrial formate
$_{c}$gly	cytosolic glycine
$_{c}$ser	cytosolic serine
$_{c}$COO	cytosolic formate
dmg	dimethylglycine
src	sarcosine

Table S3: Names of constants (μM).

GAR	10	glycinamide ribonucleotide
NADPH	50	nicotinamide adenine dinucleotide phosphate
BET	50	betaine
HCHO	500	formaldehyde
DUMP	20	deoxyuridine monophophate
$_{b}$gly	300	blood glycine (varies in some experiments)
$_{b}$ser	150	blood serine (varies in some experiments)
$_{b}$met	30	blood methionine (varies in some experiments)
FOL	20	total cellular folate (varies in some experiments)

It is assumed that total cellular folate is equally divided between the mitochondria and the cytosol [1] and that the mitochondria occupy one quarter of the volume of the cell. Thus the total normal folate concentration in the mitochondria is 40 μM, and in the cytosol is 13.3 μM.

Part B: The Equations.

For each of the biochemical reactions indicated by a reaction arrow in Figure 1, we denote the velocity of the reaction (in μM/hr) by a capital V whose subscript is the acronym for the enzyme that catalyzes the reaction. Thus, for example, the velocity of the methionine synthase reaction is denoted by V_{MS}. Each of these velocities depends on the current values one or more of the variables (metabolite concentrations) and possibly also on one or more of the constants. Velocities of reactions that occur in both the mitochondria and cytosol wiil be distinguished by c and m, for example, V_{cSHMT} and $V_{\mathrm{m}SHMT}$.

$$\left(\frac{d}{dt}\right)\mathrm{mthf} = V_{\mathrm{mFTD}}(\mathrm{m10f}) - V_{\mathrm{mSHMT}}(\mathrm{mser}, \mathrm{mthf}, \mathrm{mgly}, \mathrm{m2cf}) - V_{\mathrm{mFTS}}(\mathrm{mthf}, \mathrm{mcoo}, \mathrm{m10f}) - V_{\mathrm{mNE}}(\mathrm{mthf}, \mathrm{HCHO}, \mathrm{m2cf}) - V_{\mathrm{GDC}}(\mathrm{mgly}, \mathrm{mthf}) - V_{\mathrm{SDH}}(\mathrm{msrc}, \mathrm{mthf}) - V_{\mathrm{DMGD}}(\mathrm{mdmg}, \mathrm{mthf})$$

$$\left(\frac{d}{dt}\right)\mathrm{m2cf} = V_{\mathrm{mSHMT}}(\mathrm{mser}, \mathrm{mthf}, \mathrm{mgly}, \mathrm{m2cf}) + V_{\mathrm{mNE}}(\mathrm{mthf}, \mathrm{HCHO}, \mathrm{m2cf}) + V_{\mathrm{GDC}}(\mathrm{mgly}, \mathrm{mthf}) - V_{\mathrm{mMTD}}(\mathrm{m2cf}, \mathrm{m1cf}) + V_{\mathrm{SDH}}(\mathrm{msrc}, \mathrm{mthf}) + V_{\mathrm{DMGD}}(\mathrm{mdmg}, \mathrm{mthf})$$

$$\left(\frac{d}{dt}\right)\mathrm{m1cf} = V_{\mathrm{mMTD}}(\mathrm{m2cf}, \mathrm{m1cf}) - V_{\mathrm{mMTCH}}(\mathrm{m1cf}, \mathrm{m10f})$$

$$\left(\frac{d}{dt}\right)\mathrm{m10f} = V_{\mathrm{mFTS}}(\mathrm{mthf}, \mathrm{mcoo}, \mathrm{m10f}) + V_{\mathrm{mMTCH}}(\mathrm{m1cf}, \mathrm{m10f}) - V_{\mathrm{mFTD}}(\mathrm{m10f})$$

$$\left(\frac{d}{dt}\right)\mathrm{cthf} = V_{\mathrm{MS}}(\mathrm{hcy}, \mathrm{5mf}) + V_{\mathrm{DHFR}}(\mathrm{dhf}, \mathrm{cNADPH}) + V_{\mathrm{cFTD}}(\mathrm{c10f}) + V_{\mathrm{PGT}}(\mathrm{c10f}, \mathrm{GARP}) + V_{\mathrm{AICART}}(\mathrm{c10f}, \mathrm{aic}) - V_{\mathrm{cFTS}}(\mathrm{cthf}, \mathrm{ccoo}, \mathrm{c10f}) - V_{\mathrm{cSHMT}}(\mathrm{cser}, \mathrm{cthf}, \mathrm{cgly}, \mathrm{c2cf}) - V_{\mathrm{mNE}}(\mathrm{mthf}, \mathrm{HCHO}, \mathrm{m2cf})$$

$$\left(\frac{d}{dt}\right)\mathrm{c2cf} = V_{\mathrm{cSHMT}}(\mathrm{cser}, \mathrm{cthf}, \mathrm{cgly}, \mathrm{c2cf}) + V_{\mathrm{mNE}}(\mathrm{mthf}, \mathrm{HCHO}, \mathrm{m2cf}) - V_{\mathrm{TS}}(\mathrm{DUMP}, \mathrm{c2cf}) - V_{\mathrm{MTHFR}}(\mathrm{c2cf}, \mathrm{cNADPH}, \mathrm{sam}, \mathrm{sah}) - V_{\mathrm{cMTD}}(\mathrm{c2cf}, \mathrm{c1cf})$$

$$\left(\frac{d}{dt}\right)\mathrm{c1cf} = V_{\mathrm{cMTD}}(\mathrm{c2cf}, \mathrm{c1cf}) - V_{\mathrm{cMTCH}}(\mathrm{c1cf}, \mathrm{c10f})$$

$$
\begin{aligned}
\left(\frac{d}{dt}\right)\mathrm{c10f} &= V_{\mathrm{cFTS}}(\mathrm{cthf},\mathrm{ccoo},\mathrm{c10f}) + V_{\mathrm{cMTCH}}(\mathrm{c1cf},\mathrm{c10f}) - V_{\mathrm{cFTD}}(\mathrm{c10f}) \\
&\quad -V_{\mathrm{ART}}(\mathrm{c10f},\mathrm{aic}) - V_{\mathrm{PGT}}(\mathrm{c10f},\mathrm{GARP})
\end{aligned}
$$

$$
\left(\frac{d}{dt}\right)\mathrm{dhf} = V_{\mathrm{TS}}(\mathrm{DUMP},\mathrm{c2cf}) - V_{\mathrm{DHFR}}(\mathrm{dhf},\mathrm{cNADPH})
$$

$$
\left(\frac{d}{dt}\right)\mathrm{5mf} = V_{MTHFR}(\mathrm{c2cf},\mathrm{NADPH},\mathrm{sam},\mathrm{sah}) - V_{\mathrm{MS}}(\mathrm{hcy},\mathrm{5mf})
$$

$$
\left(\frac{d}{dt}\right)\mathrm{aic} = V_{\mathrm{PGT}}(\mathrm{c10f},\mathrm{GARP}) - V_{\mathrm{AICART}}(\mathrm{c10f},\mathrm{aic})
$$

$$
\begin{aligned}
\left(\frac{d}{dt}\right)\mathrm{met} &= V_{\mathrm{BHMT}}(\mathrm{hcy},\mathrm{BET},\mathrm{sam},\mathrm{sah}) + V_{\mathrm{MS}}(\mathrm{hcy},\mathrm{5mf}) + V_{\mathrm{bGLYc}}(\mathrm{bmet},\mathrm{met}) \\
&\quad -V_{\mathrm{MATI}}(\mathrm{met},\mathrm{sam}) - V_{\mathrm{MATIII}}(\mathrm{met},\mathrm{sam})
\end{aligned}
$$

$$
\begin{aligned}
\left(\frac{d}{dt}\right)\mathrm{sam} &= V_{\mathrm{MATI}}(\mathrm{met},\mathrm{sam}) + V_{\mathrm{MATIII}}(\mathrm{met},\mathrm{sam}) \\
&\quad -V_{\mathrm{GNMT}}(\mathrm{sam},\mathrm{sah},\mathrm{5mf},\mathrm{cgly}) - V_{\mathrm{DNMT}}(\mathrm{sam},\mathrm{sah})
\end{aligned}
$$

$$
\left(\frac{d}{dt}\right)\mathrm{sah} = V_{\mathrm{GNMT}}(\mathrm{sam},\mathrm{sah},\mathrm{5mf},\mathrm{cgly}) + V_{\mathrm{DNMT}}(\mathrm{sam},\mathrm{sah}) - V_{\mathrm{SAAH}}(\mathrm{sah},\mathrm{hcy})
$$

$$
\begin{aligned}
\left(\frac{d}{dt}\right)\mathrm{hcy} &= V_{\mathrm{SAAH}}(\mathrm{sah},\mathrm{hcy}) - V_{\mathrm{CBS}}(\mathrm{hcy},\mathrm{sam},\mathrm{sam},\mathrm{cser}) - V_{\mathrm{BHMT}}(\mathrm{hcy},\mathrm{BET},\mathrm{sam},\mathrm{sah}) \\
&\quad -V_{\mathrm{MS}}(\mathrm{hcy},\mathrm{5mf})
\end{aligned}
$$

$$
\begin{aligned}
\left(\frac{d}{dt}\right)\mathrm{mgly} &= V_{\mathrm{mSHMT}}(\mathrm{mser},\mathrm{mthf},\mathrm{mgly},\mathrm{m2cf}) - 3V_{\mathrm{mGLYc}}(\mathrm{cgly},\mathrm{mgly}) - V_{\mathrm{GDC}}(\mathrm{mgly},\mathrm{mthf}) \\
&\quad +V_{\mathrm{SDH}}(\mathrm{msrc},\mathrm{mthf})
\end{aligned}
$$

$$
\left(\frac{d}{dt}\right)\mathrm{mser} = -3V_{\mathrm{mSERc}}(\mathrm{cser},\mathrm{mser}) - V_{\mathrm{mSHMT}}(\mathrm{mser},\mathrm{mthf},\mathrm{mgly},\mathrm{m2cf})
$$

$$
\left(\frac{d}{dt}\right)\mathrm{mcoo} = -3V_{\mathrm{mHCOOHc}}(\mathrm{ccoo},\mathrm{mcoo}) - V_{\mathrm{mFTS}}(\mathrm{mthf},\mathrm{mcoo},\mathrm{m10f})
$$

$$
\begin{aligned}
\left(\frac{d}{dt}\right)\mathrm{cgly} &= V_{\mathrm{bGLYc}}(\mathrm{bgly},\mathrm{cgly}) + V_{\mathrm{cSHMT}}(\mathrm{cser},\mathrm{cthf},\mathrm{cgly},\mathrm{c2cf}) + V_{\mathrm{mGLYc}}(\mathrm{cgly},\mathrm{mgly}) \\
&\quad -V_{\mathrm{GNMT}}(\mathrm{sam},\mathrm{sah},\mathrm{5mf},\mathrm{cgly})
\end{aligned}
$$

$$\left(\frac{d}{dt}\right)\text{cser} = V_{\text{bSERc}}(\text{bser}, \text{cser}) + V_{\text{mSERc}}(\text{cser}, \text{mser}) - V_{\text{cSHMT}}(\text{cser}, \text{cthf}, \text{cgly}, \text{c2cf}) - V_{\text{CBS}}(\text{hcy}, \text{sam}, \text{sam}, \text{cser}) - (1.2)\text{cser}$$

$$\left(\frac{d}{dt}\right)\text{ccoo} = V_{\text{mHCOOHc}}(\text{ccoo}, \text{mcoo}) - V_{\text{cFTS}}(\text{cthf}, \text{ccoo}, \text{c10f})$$

$$\left(\frac{d}{dt}\right)\text{dmg} = V_{\text{BHMT}}(\text{hcy}, \text{BET}, \text{sam}, \text{sah}) - V_{\text{DMGD}}(\text{mdmg}, \text{mthf})$$

$$\left(\frac{d}{dt}\right)\text{src} = V_{\text{GNMT}}(\text{sam}, \text{sah}, \text{5mf}, \text{cgly}) + V_{\text{DMGD}}(\text{mdmg}, \text{mthf}) - V_{\text{SDH}}(\text{msrc}, \text{mthf})$$

The velocities of transport from blood to cytosol or mitochondria to cytosol are given by the transparent notation V_{bSERc} and V_{mSERc}, respectively. The units are in μM/hr increase *in the cytosol.* Since the cytosol is assumed to have three times the volume of the mitochondria, one μM increase in the cytosol due to transport from the mitochondria means a 3 μM decrease in the mitochondria. This is the reason for the 3's in the equations involving transport into and out of the mitochondria and in the transport kinetics below.

Part C: Kinetics.

For many of the velocities, we assume that their dependence on substrates has Michaelis-Menten form with one substrate

$$V = \frac{V_{max}[S]}{K_m + [S]},$$

or random order Michaelis-Menten form with two substrates:

$$V = \frac{V_{max}[S_1][S_2]}{(K_{m,1} + [S_1])(K_{m,2} + [S_2])}.$$

Some reactions, for example V_{cSHMT}, are assumed to have reversible random order Michaelis-Menten kinetics with two substrates in each direction. For all these velocities, the form of the kinetics is clear. The K_m and V_{max} values appear in Table S4 (modified from [2]), along with references. Enzymes that occur in both the mitochondria and the cytosol are assumed to have the same kinetics in both compartments. If the kinetic constants differ in the cytosol and the mitochondria that is indicated by the prefixes m and c, respectively. Reactions that do not have one of these simple forms and transport velocities are discussed individually after Table S4.

Table S4. Model kinetic parameter values (time in hrs., concentrations in μM).

Parameter	Literature	Model	Reference
**AICART**			
$K_{\mathrm{m,10f}}$	5.9-50	5.9	[3][4][5][6]
$K_{\mathrm{m,aic}}$	10-100	100	[3][4][6]
V_{max}	370-44400	55000	
**DHFR.**			
$K_{\mathrm{m,dhf}}$	0.12-1.9	0.5	[7][3][4][8]
$K_{\mathrm{m,NADPH}}$	0.3-5.6	4.0	[7][3][4][8]
V_{max}	350-23000	2000	[7][3][4]
**DMGD**			
$K_{\mathrm{m,dmg}}$	50	50	[9]
$K_{\mathrm{m,mthf}}$	-	50	
V_{max}	-	15000	
**FTD.**			
$K_{\mathrm{m,10f}}$	0.9-20	20	[10][11]
${}_{\mathrm{c}}V_{max}$		500	
${}_{\mathrm{m}}V_{max}$		1050	
**cFTS.**			
$K_{\mathrm{m,thf}}$	0.1 - 600	3	[3][5]
$K_{\mathrm{m,coo}}$	8 - 1000	43	[3][5]
V_{max}	100 - 486000	3900	[3][5]
**mFTS.**			
$K_{\mathrm{m,thf}}$	0.1 - 600	3	[3][5]
$K_{\mathrm{m,coo}}$	8 - 1000	43	[3][5]
V_{max}	100 - 486000	2000	[3][5]
$K_{\mathrm{m,10f}}$	-	22	
V_{max}	-	6300	
**GDC**			
$K_{\mathrm{m,mgly}}$	3400-40000	3400	[15][12][13][14]
$K_{\mathrm{m,mthf}}$	50	50	[16]
V_{max}	-	15000	
**MS.**			
$K_{\mathrm{m,5mf}}$	22-34	25	[17][18]
$K_{\mathrm{m,hcy}}$	0.1-6	1	[19]
V_{max}		500	[19]

Parameter	Literature	Model	Reference
**MTCH.** (positive direction is from 1cf to 10f)			
$K_{\mathrm{m,1cf}}$	4-250	250	[3][4][5]
cV_{max}	880-1380000	500000	[4][5]
mV_{max}	880-1380000	790000	[4][5]
$K_{\mathrm{m,10f}}$	20-450	100	[3][4][5]
V_{max}	10.5-1380000	20000	[4][5]
**MTD.** (positive direction is from 2cf to 1cf)			
$K_{\mathrm{m,2cf}}$	2-5	2	[3][5]
cV_{max}	520-594000	80000	[7][3][5]
mV_{max}	520-594000	180000	[7][3][5]
$K_{\mathrm{m,1cf}}$	1-10	10	[20][5]
V_{max}	594000	600000	[5]
**PGT.**			
$K_{m,\mathrm{10f}}$	4.9-58	4.9	[3][4][21][22]
$K_{\mathrm{m,GAR}}$	520	520	[3][4][21][22]
V_{max}	6600-16200	24300	[3][4][21][22]
**SAHH.** (positive direction is from sah to hcy)			
$K_{\mathrm{m,sah}}$		10	[23]
V_{max}		5000	[23]
$K_{\mathrm{m,hcy}}$		1	[23]
V_{max}		5000	[23]
**SDH**			
$K_{\mathrm{m,dmg}}$	320	320	[24]
$K_{\mathrm{m,mthf}}$	-	50	
V_{max}	-	15000	
**SHMT.** (positive direction is from thf to 2cf)			
$K_{\mathrm{m,ser}}$	350-1300	600	[3][4][5][25][26]
$K_{\mathrm{m,thf}}$	45-300	50	[3][4][5][27][28]
cV_{max}	500-162000	5200	[7][4][5][28]
mV_{max}	500-162000	11440	[7][4][5][28]
$K_{\mathrm{m,gly}}$	3000-10000	10000	[7][3][4][5][26]
$K_{\mathrm{m,2cf}}$	3200-10000	3200	[7][4][5][27]
cV_{max}	12600-120000000	15000000	[7][4][5]
mV_{max}	12600-120000000	30000000	[7][4][5]
**TS.**			
$K_{\mathrm{m,DUMP}}$	5-37	6.3	[7][3][29][30]
$K_{\mathrm{m,2cf}}$	10-45	14	[7][3][29][30]
V_{max}	30-4200	5000	[4][30]

We now discuss the reactions where the kinetics have a special form.

BHMT. The kinetics of BHMT are Michaelis-Menten with the parameters $K_{m,1} = 12$, $K_{m,2} = 100$, and $V_{max} = 2160$ [31],[32]. The form of the inhibition of BHMT by SAM was derived by non-linear regression on the data of [33] and scaled so that it equals 1 when the external methionine concentration is 30 μM.

$$V_{\text{BHMT}} = e^{-.0021(\text{sam}+\text{sah})}e^{+.0021(76.7)}\frac{V_{max}(\text{hcy})(\textsc{bet})}{(K_{m,1}+\text{hcy})(K_{m,2}+\textsc{bet})}$$

The inhibition of BHMT is controversial because Bose et al. [34] found that sam has no effect on recombinant BHMT's ability to remethylate homocysteine. It is possible that sam affects the expression of BHMT rather than BHMT itself in which case the results of [33] and [34] would not be contradictory. In any case, the inhibition that we use has significant effects on the BHMT reaction only for sam concentrations well above normal. Thus, only the methionine and protein loading experiments would be affected by removing the inhibition of BHMT by hcy, in which case hcy does not rise as much during loading (simulations not shown). Similarly, we found in [23] that the presence or absence of this inhibition had little effect on the stabilization of DNA methylation.

CBS. The kinetics of CBS are standard Michaelis-Menten with $K_{m,1} = 1000$ for hcy taken from [35] and $K_{m,2} = 2000$ for cser taken from [36], with $V_{max} = 402,000$. The form of the activation of CBS by sam and sah was derived by non-linear regression on the data in [37] and [38] and scaled so that it equals 1 when the external methionine concentration is 30 μM.

$$V_{\text{CBS}} = \left(\frac{V_{max}(\text{hcy})(\text{cser})}{(K_m+\text{hcy})(K_m+\text{cser})}\right)\left(\frac{(1.2)(\text{sam}+\text{sah})^2}{(30)^2+(\text{sam}+\text{sah})^2}\right).$$

DNMT. The DNA methylation reaction is given as a uni-reactant scheme with sam as substrate. That is, the substrates for methylation are assumed constant. Their variation can be modeled by varying the V_{max}. The kinetic constants, $V_{max} = 180$, $K_m = 1.4$, and $K_i = 1.4$ are from [39].

$$V_{\text{DNMT}} = \frac{V_{max}(\text{sam})}{K_m(1+\frac{\text{sah}}{K_i})+\text{sam}}.$$

GNMT. The first factor of the GNMT reaction is standard Michaelis-Menten with $V_{max} = 245$, and $K_{m,1} = 63$ for sam and $K_{m,2} = 130$ for cgly estimated from [41]. The second term is product inhibition by sah from [40] with $K_i = 18$. The third term, the long-range inhibition

of GNMT by 5mf, was derived by non-linear regression on the data of [42], Figure 3, and scaled so that it equals 1 when the external methionine concentration is 30 μM.

$$V_{\text{GNMT}} = \left(\frac{V_{max}(\text{sam})(\text{cgly})}{(K_{m,1}+\text{sam})(K_{m,2}+\text{cgly})}\right)\left(\frac{1}{1+\frac{\text{sah}}{K_i}}\right)\left(\frac{5.88}{0.35+5\text{mf}}\right)$$

MAT-I. The MAT-I kinetics are from [43], Table 1, and we take $V_{max} = 260$ and $K_m = 41$. The inhibition by sam was derived by non-linear regression on the data from [43], Figure 5.

$$V_{\text{MAT-I}} = \left(\frac{V_{max}(\text{met})}{K_m+\text{met}}\right)(0.23+(0.8)e^{-(0.0026)(\text{sam})})$$

MAT-III. The methionine dependence of the MAT-III kinetics is from [44], Figure 5, fitted to a Hill equation with $V_{max} = 220$, $K_m = 300$. The activation by sam is from [43], Figure 5, fitted to a Hill equation with $K_a = 360$.

$$V_{\text{MAT-III}} = \left(\frac{V_{max}(\text{met})^{1.21}}{K_m+(\text{met})^{1.21}}\right)\left(1+\frac{(7.2)(\text{sam})^2}{(K_a)^2+(\text{sam})^2}\right)$$

MTHFR. The first factor in the formula for the MTHFR reaction velocity

$$V_{\text{MTHFR}} = \frac{V_{max}(2\text{cf})(\text{NADPH})}{(K_{m,1}+2\text{cf})(K_{m,2}+\text{NADPH})}\cdot\frac{60}{10+\text{sam}-\text{sah}}$$

is standard Michaelis-Menten with $K_{m,1} = 50$, $K_{m,2} = 16$, and $V_{max} = 7000$ taken from [45][46][25].

The inhibition of MTHFR by sam, the second factor, was derived by non-linear regression on the data of [47][48] and has the form $10/(10+\text{sam})$. In addition, sah competes with sam for binding to the regulatory domain of MTHFR. It neither activates nor inhibits the enzyme [48] but prevents inhibition by sam; thus, we take our inhibitory factor to be:

$$I = \frac{10}{10+\text{sam}-\text{sah}}.$$

The factor 60 scales the inhibition so that it has value 1 when the external methionine concentration is 30 μM.

NE. The kinetics of the non-enzymatic reversible reaction between thf and 2cf are taken to be mass action,

$$V_{\text{NE}} = k_1(\text{thf})(\textsc{hcho}) - k_2(\text{2cf}).$$

The rate constants are $k_1 = 0.03$, and $k_2 = 22$ in the cytosol and $k_2 = 20$ in the mitochondria.

We now discuss the kinetics of transport between the compartments. The general formula for the kinetics of transport between the blood and the cytosol is taken to be

$$V = \frac{V_{max}[bAA]}{K_m + [bAA]} - k_{\text{out}}[cAA],$$

where AA stands for an amino acid and the prefixes b and c refer to the blood and cytosolic compartments, respectively. Thus the kinetics are Michaelis-Menten coming into the cell and linear going out. We take the kinetics of transport of serine and glycine between the cytosol and mitochondria to be Michaelis-Menten in both directions and the transport of formate between these two compartments to be linear in both directions.

Transport of amino acids into cells is accomplished by a relatively small number of transport systems each of which handles several amino acids. Each transporter is specialized to handle amino acids with particular ionic characteristics [49][50]. The transporters are saturable and the K_m values have been measured in many systems [51][52][53]. Relatively little is known about the kinetics by which amino acids leak out of cells so we take this process to be linear. This linear rate also includes the loss of cytosolic amino acids to other metabolic processes not in the model (see Figure 1), for example use in protein synthesis.

Table S5. Parameter values for transporters (hours, μM).

Parameter	Model value	Parameter	Model value
V_{bGLYc}		V_{mGLYc}	
K_m	150	$K_{m,\text{cgly}}$	5700
V_{max}	2000	V_{max}	10000
k_{out}	1	$K_{m,\text{mgly}}$	5700
		V_{max}	10000/3
V_{bSERc}		V_{mSERc}	
K_m	150	$K_{m,\text{cser}}$	5700
V_{max}	2700	V_{max}	10000
k_{out}	1	$K_{m,\text{mser}}$	5700
		V_{max}	10000/3
V_{bMETc}		V_{mHCOOHc}	
K_m	150	k_{in}	100/3
V_{max}	910	k_{out}	100
k_{out}	1		

Part C: *In silico* experimentation.

If one starts with any initial values for the variables and solves the differential equations when the velocities have the formulas given in Part C, one finds that the variables all approach steady concentrations after a few hours. The concentrations of the variables and the velocities of reactions at this "normal" steady state are given in Table 1 in the main body of the paper. Most of the *in silico* experiments reported in the paper were done by starting the system at this steady state, changing one or more parameters, and letting the system relax to a new steady state. For example, in Section A of Results, the external glycine concentration, bgly, was varied systematically from 50 to 900 μM, and for each such concentration the resulting values of various velocities and metabolite concentrations were computed at steady state. In Section B of Results, the total cellular folate, FOL, was changed from 20 μM to 10 μM. By solving the differential equations we could report the effect of this change on velocities and metabolite concentrations. Similarly, the effect of changing the expression of cSHMT was computed by changing the V_{max} of the SHMT reaction and computing the new steady state. In other experiments, reactions, or whole groups of reactions, were removed entirely. Finally, in the methionine loading and protein loading experiemnts, the blood concentrations of glycine, serine, and methionine were allowed to vary in time. By solving the differential equations we saw how the various velocities and metabolite concentrations varied in time.

References

[1] Cook, R. J. (2001) **Folate Metabolism**. In *Homocysteine in Health and Disease*, eds. Carmel, R., and Jacobsen, D. W., Cambridge Univ. Press, Cambridge.

[2] Nijhout, H. F., Reed, M. C., Budu, P., and Ulrich, C. M. 2004. **A Mathematical Model of the Folate Cycle.** *J. Biol. Chem.* **279**, 55008-55016

[3] Vorontzov, I. N., Greshilov, M. M., Belousova, A. K., and Gerasimova, G.K. (1980)**Mathematical description and investigation of the principles of functioning of the folic acid cycle.** *Biokhimiya*, **45**, 83-97.

[4] Seither, R. L., Trent, D. F., Mikulecky, D. C., Rape, T. J., and I. D. Goldman, I. D. (1989) **Folate-pool interconversions and inhibition of biosynthetic processes after exposure of L1210 leukemia cells to antifolates.** *J. Biol. Chem.*, **264**, 17016-17023.

[5] Strong, W. B., Tendler, S. J., Seither, R. L.., Goldman, I. D., and Schirch, V. (1990) **Purification and properties of serine hydroxymethyltransferase and C1-tetrahydrofolate synthase from L1210 cells,** *J. Biol. Chem.*,**265**,

[6] Rayl, E.A., Moroson, B.A. and Beardsley, G.P. (1996) **The human purH gene product, 5-aminoimodazole-4-carboxamide ribonucleotide formyltransferase/IMP**

cyclohydrolase. Cloning, sequence, expression, purification, kinetic analysis, and domain mapping, *J. Biol. Chem.*,**271**, 2225-2233.

[7] Jackson, R.C. and Harrap, K.R. (1973) **Studies with a mathematical model of folate metabolism,** *Arch. Biochem. Biophys.*, **158**, 827-841.

[8] Blakley, R. L. (1995) **Eukaryotic dihydrofolate reductase,** *Adv. Enzymol.* **60**, 23-102.

[9] Porter, D. H., Cook, R. J., and Wagner, C. (1985) **Enzymatic properties of dimethylglycine dehydrogenase and sarcosine dehydrogenase from rat liver**, *Arch. Biochem. Biophys.*, **243**, 396-407.

[10] Schirch, D., Villar, E., Maras, B., Barra, D. and Schirch, V. (1994) Domain structure and function of 10-formyltetrahydrofolate dehydrogenase, *J. Biol. Chem.* **269**, 24728 - 24735.

[11] Kim, D. W., Huang, T., Schirch, D., and Schirch, V. (1996) **Properties of Tetrahydropteroylpentaglutamate bound to 10-formyltetrahydrofolate dehydrogenase**, *Biochem.*,**35**, 15772-15783.

[12] Hiraga, K., and Kikuchi, G. (1980) **The mitochondrial glycine cleavage system: purification and properties of glycine decarboxylase from chicken liver mitochondria**, *J. Biol. Chem.*, **255**, 11664-11670.

[13] Gueguen, V., Macherel, D., Neuberger, M., Saint Pierre, C., Jaquinod, M., Gans, P., Douce, R., and Bourguignon, J. (1999) **Structural and functional characterization of H protein mutants of the glycine decarboxylase complex**, *J. Biol. Chem.*, **274**, 26344-26352.

[14] Fujiwara, K., and Motokawa, Y. (1983) **Mechanism of the glycine cleavage reaction: steady state kinetic studies of the P-protein-catalyzed reaction**, *J. Biol. Chem.*, **258**, 8156-8162.

[15] Verleysdonk, S., Martin, H., Willker, W., Leibfritz, D., and Hamprecht, B. (1999) **Rapid uptake and degradation of glycine by astroglial cells in culture: synthesis and release of serine and lactate**, *Glia*, **27**, 239-248.

[16] Fujiwara, K., Okamura-Ikeda, K., and Motokawa, Y. (1984) **Mechanism of the glycine cleavage reaction: further characterization of the intermediate attached to the H-protein and of the reaction catalyzed by the T-protein**, *J. Biol. Chem.*, **259**, 10664-10668.

[17] Finkelstein, J. D., and Martin, J. J. (1986) **Methionine metabolism in mammals: adaptation to methionine excess**, *J. Biol. Chem.* **261**, 1582-1587.

[18] Banerjee, R., Frasca, V., Ballou, D., and, Matthews, R., 1990. **Participation of Cob(I)alamin in the reaction catalyzed by methionine synthase from *Escherichia coli*: a steady state and rapid reaction kinetic analysis**, *Biochem.*, **29**, 11101-11109.

[19] Banerjee, R., Chen, Z., and Gulati, S. 1997. **Methionine synthase from pig liver**, *Meth. Enzymol.*, **281**, 189-197.

[20] Wagner, C. (1995) **Biochemical role of folate in cellular metabolism**, in *Folate in Health and Disease*(Bailey, L. B., ed.), pp. 23-42, Marcel Dekker, New York.

[21] Caperelli, C.A. (1985) **Mammalian glycinamide ribonucleotide transformylase: purification and some properties**, *Biochemistry*,**24**, 1316-1320.

[22] Caperelli, C.A. (1989) Mammalian glycinamide ribonucleotide transformylase. Kinetic mechanism and associated de novo purine biosynthetic activities. *J. Biol. Chem.*,**264**, 5053-5057.

[23] Nijhout, H. F., Reed, M. C., Anderson, D. A., Mattingly, J., James, S. J., Ulrich, C. M. (2006) **Long-Range Allosteric Interactions between the Folate and Methionine Cycles Stabilize DNA Methylation Reaction Rate**, *Epigenetics*, **1**, 81-87.

[24] Frisell, W. R., and Mackenzie, C. G. (1970) **Sarcosine dehydrogenase and dimethylglycine dehydrogenase (rat liver, monkey liver)**, *Methods Enzymol.*, **17A**, 976-981.

[25] Daubner, S. C. and Matthews, R. G. (1982) **Purification and properties of methylenetetrahydrofolate reductase from pig liver**,*J. Biol. Chem.*,**257**, 140-145.

[26] Schirch, V., Hopkins, S., Villar, E. and Angelaccio, S. (1985) **Serine hydroxymethyltransferase from Escherichia coli: purification and properties**, *J. Bacteriol.*,**163**, 1-7.

[27] Schirch, V. (1997) **Purification and properties of folate-dependent enzymes from rabbit liver**, *Meth. Enzymol.*,**281**, 146-161.

[28] Schirch, L. and Peterson, D. (1980) **Purification and properties of mitochondrial serine hydroxymethyltransferase**, *J. Biol. Chem.*,**255**, 7801-7806.

[29] Carreras, C. W. and Santi, D. V. (1995) **The catalytic mechanism and structure of thymidylate synthase**, *Ann. Rev. Biochem.*,**64**, 721-762.

[30] Lu, Y.-Z., Aiello, P. D. and Matthews, R.G. (1984) **Studies on the polyglutamate specificity of thymidylate synthase from fetal pig liver**, *Biochemistry*, **23**, 6870-6876.

[31] Finkelstein, J. D., Harris, B. J., and Kyle, W. E. (1972) **Methionine metabolism in mammals: kinetic study of betaine-homocysteine methyltransferase**, *Arch. Biochem. Biophys.*, **153**, 320-324.

[32] Skiba, W. E., Taylor, M. P., Wells, M. S., Mangum, J. H., and Awad, W. M. (1982) **Human hepatic methionine biosynthesis. Purification and characterization of betaine:homocysteine S-methyltransferase**, *J. Biol. Chem.*, **257**, 14944-14948.

[33] Finkelstein, J. D., and Martin, J. J. (1984) **Inactivation of betaine-homocysteine methyltransferase by adenosylmethionine and adenosylethionine**, *Biochem. Biophys. Res. Comm.* **118**, 14-19.

[34] Bose, N., Greenspan, P., and Momany, C. (2002) **Expression of recombinant human betaine:homocysteine S-methyltransferase for X-ray crystallographic studies and further characterization of interaction with S-andenosylmethionine**, *Prot. Exp. Purif.*, **25**, 73-80.

[35] Finkelstein, J. (2001) **Regulation of homocysteine metabolism.** In *Homocysteine in Health and Disease* (Carmel, R., and Jacobsen, D. W., eds) pp. 92-99, Cambridge Univ. Press, Cambridge.

[36] Taoka S., Ohja, S., Shan, X., Kruger, W. D., and Banerjee, R. (1998) **Evidence for heme-mediated redox regulation of human cystathionine beta-synthase activity**, *J. Biol. Chem.*, **273**, 25179-25184.

[37] Janosik, M., Kery, V., Gaustadnes, M., Maclean, K. N., and Kraus, J. P. (2001) **Regulation of Human Cystathionine -Synthase by S-Adenosyl-L-methionine: Evidence for Two Catalytically Active Conformations Involving an Autoinhibitory Domain in the C-Terminal Region**, *Biochemistry* **40**, 10625-10633

[38] Kluijtmans, L. A. J., Boers, G. H. J., Stevens, E. M. B., Renier, W. O., Kraus, J. P., Trijbels, F. J. M., van den Heuvel, L. P. W. J., and Blom, H. J. (1996) **Defective cystathionine beta-synthase regulation by S-adenosylmethionine in a partially pyridoxine responsive homocystinuric patient**, *J. Clin Invest.* **98**, 285-289.

[39] Flynn, J., and Reich, N. **Murine DNA (Cytosine-5-)-methyltransferase: Steady-State and Substrate Trapping Analyses of the Kinetic Mechanism**, (1998) *Biochemistry* **37**, 15162-15169

[40] Reed, M. C., Nijhout, H. F., Sparks, R., and Ulrich, C. M. (2004) **A Mathematical model of the methionine cycle,** *J. Theor. Biol.* **226**, 33-43.

[41] Ogawa, H., and Fujioka, M. (1982) **Purification and properties of glycine N-methyltransferase from rat liver**, *J. Biol. Chem.* **257**, 3447-3452

[42] Yeo, E.-J., and Wagner, C. (1992) **Purification and properties of pancreatic glycine N-methyltransferase**, *J. Biol. Chem.* **267**, 24669-24674

[43] Sullivan, D. M., and Hoffman, J. L. (1983) **Fractionation and kinetic properties of rat liver and kidney methionine adenosyltransferase isozymes**, *Biochemistry* **22**, 1636-1641.

[44] Sanchez del Pino, M. M., Corrales, F. J., and Mato, J. M. (2000) **Hysteretic Behavior of Methionine Adenosyltransferase III. Methionine switches between two conformations of the enzyme with different specific activity**, *J. Biol. Chem.* **275**, 23476-23482

[45] Matthews, R.G. (1986) **Methylenetetrahydrofolate reductase from pig liver,** *Meth. Enzymol.*,**122**, 372-381.

[46] Green, J. M., MacKenzie, R. E., Matthews, R. G. (1988) **Substrate flux through methylenetetrahydrofolate dehydrogenase: Predicted effects of the concentration of methylenetetrahydrofolate on its partitioning into pathways leading to nucleotide biosynthesis or methionine regeneration**, *Biochemistry* **27**, 8014-8022.

[47] Jencks, D. A., and Matthews, R. G. (1987) **Allosteric inhibition of methylenetetrahydrofolate reductase by adenosylmethionine. Effects of adenosylmethionine and NADPH on the equilibrium between active and inactive forms of the enzyme and on the kinetics of approach to equilibrium**, *J. Biol. Chem.* **262**, 2485-2493.

[48] Yamada, K., Chen, Z., Rozen, R., and Matthews, R. G. (2001) **Effects of common polymorphisms on the properties of recombinant human methylenetetrahydrofolate reductase**, *PNAS* **98**, 14853-14858.

[49] Palacin, M., Estevez, R., Bertran, J., and Zorzano, A. (1998) **Molecular biology of mammalian plasma membrane amino acid transporters**, *Physiol. Rev.*, **78**, 969-1054.

[50] Mailliard, M. E., and Kilberg, M. S. (1990) **Sodium-dependent neutral amino acid transport by human liver plasma membrane vesicles**, *J. Biol. Chem.*, **265**, 14321-14326.

[51] Kilberg, M. S. and Haeussinger, D. (1992) *Mammalian Amino Acid Transport: Mechanisms and Control*, Plenum Pr., New York

[52] Soriano-Garcia, J. F., Torras-Llort, M., Ferrer, R., and Moreto, M. (1998) **Multiple pathways for L-methionine transport in brush-border membrane vesicles from chicken jejunum**, *J. Physiol.*, **509.2**, 527-539.

[53] Boerner, P., and Saier, M. H. (1982) **Growth regulation and amino acid transport in epithelial cells: influence of culture conditions and transformation on A,ASC, and I transport activities**, *J. Cell Physiol.*, **113**, 240-246.
